# In vivo diffusion MRI of the human heart using a 300 mT/m gradient system

**DOI:** 10.1002/mrm.30118

**Published:** 2024-04-22

**Authors:** Maryam Afzali, Lars Mueller, Sam Coveney, Fabrizio Fasano, Christopher John Evans, Maria Engel, Filip Szczepankiewicz, Irvin Teh, Erica Dall’Armellina, Derek K. Jones, Jürgen E. Schneider

**Affiliations:** 1 Biomedical Imaging Science Department, Leeds Institute of Cardiovascular and Metabolic Medicine, University of Leeds, Leeds, UK; 2 Cardiff University Brain Research Imaging Centre (CUBRIC), Cardiff University, Cardiff, UK; 3 Siemens Healthcare Ltd, Camberly, UK; 4 Siemens Healthcare GmbH, Erlangen, Germany; 5 Medical Radiation Physics, Clinical Sciences Lund, Lund University, Lund, Sweden

**Keywords:** cardiac diffusion MRI, higher-order motion compensation, strong gradients

## Abstract

**Purpose:**

This work reports for the first time on the implementation and application of cardiac diffusion-weighted MRI on a Connectom MR scanner with a maximum gradient strength of 300 mT/m. It evaluates the benefits of the increased gradient performance for the investigation of the myocardial microstructure.

**Methods:**

Cardiac diffusion-weighted imaging (DWI) experiments were performed on 10 healthy volunteers using a spin-echo sequence with up to second- and third-order motion compensation (*M*_2_ and *M*_3_) and *b* = 100, 450, and 1000 s/mm^2^ (twice the *b*_max_ commonly used on clinical scanners). Mean diffusivity (MD), fractional anisotropy (FA), helix angle (HA), and secondary eigenvector angle (E2A) were calculated for *b* = [100, 450] s/mm^2^ and *b* = [100, 1000] s/mm^2^ for both *M*_2_ and *M*_3_.

**Results:**

The MD values with *M*_3_ are slightly higher than with *M*_2_ with ΔMD = 0.05 ± 0.05 [×10^−3^ mm^2^/s] (*p* = 4*e* − 5) for *b*_max_ = 450 s/mm^2^ and ΔMD = 0.03 ± 0.03 [× 10^−3^ mm^2^/s] (*p* = 4*e* − 4) for *b*_max_ = 1000 s/mm^2^. A reduction in MD is observed by increasing the *b*_max_ from 450 to 1000 s/mm^2^ (ΔMD = 0.06 ± 0.04 [× 10^−3^ mm^2^/s] (*p* = 1.6*e* − 9) for *M*_2_ and ΔMD = 0.08 ± 0.05 [× 10^−3^ mm^2^/s] (*p* = 1*e* − 9) for *M*_3_). The difference between FA, E2A, and HA was not significant in different schemes (*p* > 0.05).

**Conclusion:**

This work demonstrates cardiac DWI in vivo with higher *b*-value and higher order of motion compensated diffusion gradient waveforms than is commonly used. Increasing the motion compensation order from *M*_2_ to *M*_3_ and the maximum *b*-value from 450 to 1000 s/mm^2^ affected the MD values but FA and the angular metrics (HA and E2A) remained unchanged. Our work paves the way for cardiac DWI on the next-generation MR scanners with high-performance gradient systems.

## 1 Introduction

Diffusion MRI sensitizes the MR signal to the random motion of water molecules in tissue.^1^ By probing the water motion in tissue, one can infer information about the underlying microstructure.^2^ The heart is arguably one of the most challenging organs for diffusion MRI because of cardiac and respiratory motion. Macroscopic motion can cause significant signal loss and therefore dedicated motion-compensation techniques are required to scan the beating heart.^3,4^

Cardiac diffusion-weighted MR images can be obtained free breathing using a spin-echo (SE) sequence^5^ which allows the acquisition within one cardiac cycle. However, SE cardiac diffusion-weighted imaging (DWI) with traditional monopolar encoding is sensitive to cardiac motion which changes phase coherence and can be mistaken for diffusion. One solution to this problem is to use motion-compensated diffusion-encoding gradient waveforms that are sensitive to diffusion motion and insensitive to bulk motion.^3^

Nulling the first moment of diffusion gradients (*M*_1_-nulling) to achieve velocity-compensation was first introduced for SE cardiac DWI by Gamper et al.^3^ Throughout this paper, *M*_*n*_ means all moments lower than n are also nulled. More recently, diffusion gradients with moments nulled up to the second order (*M*_2_-nulling), providing both velocity and acceleration compensation, have been used.^6,4,7^ Velocity and acceleration-compensated methods are presently the most common approach for SE cardiac DWI in human hearts where echo times (TEs) of 65−77 ms at a maximum *b*-value of 350–500 s/mm^2^ (maximum gradient strength of 80 mT/m) have been reported.^8–10^

The total duration of any moment-nulled gradient waveform is longer than the one of monopolar waveforms. As a result, motion-compensated waveforms increase the TE which reduces the signal-to-noise-ratio (SNR). Up to third-order motion compensation, that is, jerk-nulling (*M*_3_-nulling), has so far only been explored in rat hearts on a preclinical MRI scanner.^11^

Unsurprisingly, most of the in vivo human, second-order motion compensated (*M*_2_) SE cardiac DWI studies so far are limited to a maximum *b*-value of 500 s/mm^2^ to keep the TE within a reasonable range (TE < 80 ms). However, by increasing the *b*-value the sensitivity of the diffusion-weighted signal to smaller spatial scales increases,^12^ which allows to probe tissue microstructure of the heart.^13^ The advantage of using higher *b*-values is shown in several cardiac DWI ex vivo studies.^13,14^ The work from Teh et al.^15^ on in vivo human heart suggested that higher *b*-values may be advantageous in inferring microstructural information from cardiac tissue.

Improved gradient performance allows for shorter diffusion gradient waveforms (at a given *b*-value) and, therefore, shorter TEs, which in turn reduces signal losses from T2 relaxation.^16–19^ The Connectom MR system^16,17^ features magnetic field gradients four to eight times stronger than those on commonly available clinical MR scanners. The Connectom MR scanner has significantly reduced the resolution limit for axon diameter estimation in the brain or drastically improved the microstructural anisotropy assessment (see Reference 20 for details). Thus, similar to these neuro applications, the increased gradient performance can be expected to give new insights into cardiac microstructure. This scanner therefore also represents an opportunity to investigate the effect of higher-order motion compensation in the human heart in vivo.

Here, we demonstrate cardiac DWI investigations of the human heart on a Connectom MR system. In addition, we sought to establish the feasibility of applying third-order motion-compensated (*M*_3_) diffusion gradients at *b*_max_ = 1000 s/mm^2^, which would result in prohibitively long TEs in conventional clinical MR scanners. The main focus of this work is to compare motion compensations up to second versus up to third-order (*M*_2_ vs. *M*_3_) and low versus high *b*-value (*b*_max_ = 450 s/mm^2^ vs. *b*_max_ = 1000 s/mm^2^).

## 2 Methods

### 2.1 Experimental setup and recruitment

Cardiac diffusion-weighted images were acquired on a Connectom 3T research-only MR imaging system (Siemens Healthcare) with a maximum gradient strength of 300 mT/m and slew rate of 200 T/m/s. An 18-channel body receive coil was used in combination with a 32-channel spine receive coil. Ten healthy (no known previous cardiac conditions) volunteers were recruited for this study (age range 19−36 years old (24.1 ± 6.4 years old), weight range of 54.9−112 kg (72.6 ± 20.5 kg), six females and four males). The studies were approved by the Cardiff University School of Psychology Ethics Committee and all subjects provided written consent.

### 2.2 Gradient waveform design

Traditional motion-compensated diffusion encoding gradient waveforms comprise trapezoidal gradient waveforms which are (anti-)symmetric around the 180° radiofrequency (RF) pulse.^3,11^ However, spin-echo echo-planar imaging (SE-EPI) introduces dead times before the refocusing pulse, therefore, it is more efficient to design asymmetric waveforms.^21–23^ Asymmetric waveforms are prone to concomitant fields^24^ which can have a severe effect on the signal. To mitigate against this confounder, we consider Maxwell compensation in our waveform design.^25^ The Maxwell compensated waveform necessitated in our case an increase of the TE by 4−6 ms (this correction may not always be essential but it becomes more important for higher gradient amplitudes since the concomitant fields scale with *G*^2^).

Diffusion gradient waveforms were designed using the NOW toolbox^26,27^ (https://github.com/jsjol/NOW) to provide Maxwell-compensated waveforms that can reach a specified *b*-value in the shortest TE.

The waveform design was performed offline. For designing the waveform, we determined—the timing before the 180° pulse, the duration of the 180° pulse, the time after the 180° pulse, *G*_max_, SR_max_, and order of motion compensation, respectively. During the optimization, the encoding time was discretized into 77 (the default value in the toolbox) timesteps (of equal length), and the gradient amplitude varied at the time points to achieve the highest *b*-value under the following constraints: (i) linear shape of the b-tensor (gradients along a single axis); (ii) slew rate of less than 80 T/m/s to remain within the MR scanner-imposed stimulation limits (see Table S1 and Figures S3, S4, and S5, for more details); (iii) maximum gradient amplitude less than 300 mT/m; (iv) Maxwell compensation^25^ (see Figure S7, for more details); and (v) the gradient moments up to the desired order should be smaller than a threshold (10^−4^ in unit of *s^n^/m*, where *n* is the motion compensation order^27^). The maximum *b*-value, in this work, was 1000 s/mm^2^, and other *b*-values were achieved by scaling the gradient amplitudes while keeping the shape and timing constant. It is more SNR efficient to design the waveforms for each *b*-value separately.

**Figure 1:**
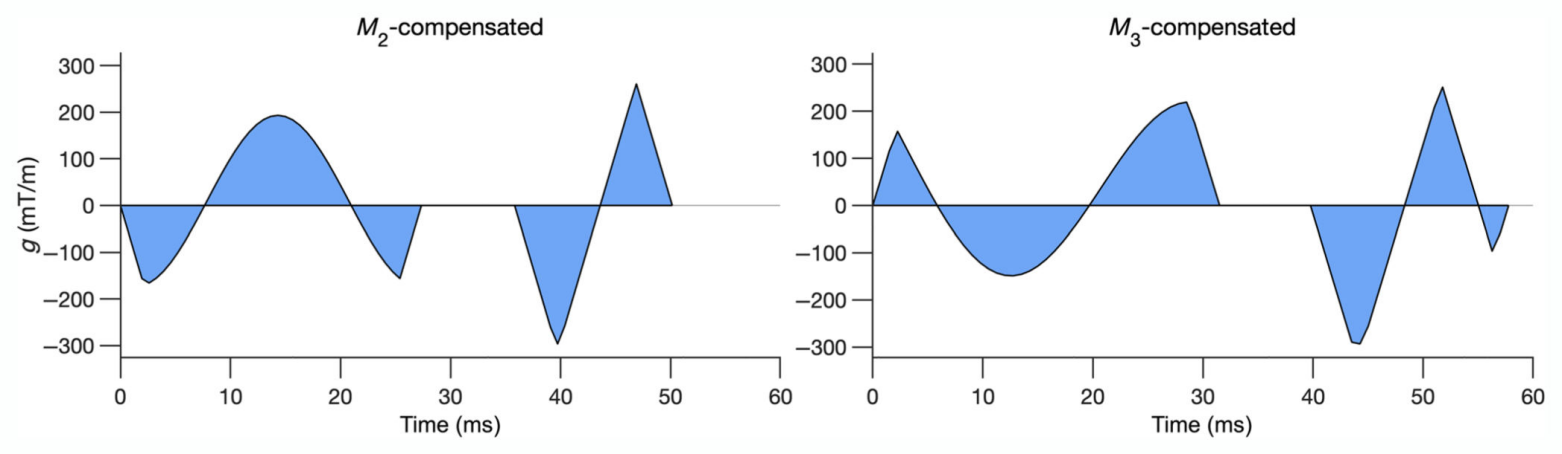
Numerically optimized motion compensated waveforms up to second- and third-order motion compensation (*M*_2_ and *M*_3_). The waveforms are optimized for *b*_max_ = 1000 s/mm^2^, *G*_max_ = 300 mT/m and maximum slew rate of 80 T/m/s (see Table S1 for more details).

However, some part of our work is focused on comparing the effect of maximum *b*-value on the estimated diffusion metrics (*b* = [100, 450] s/mm^2^ vs. *b* = [100, 1000] s/mm^2^). We therefore considered it as important to avoid any contribution from TE difference, and kept the TE constant for both *b* = [100, 450] s/mm^2^ versus *b* = [100, 1000] s/mm^2^ scenarios. The waveforms with up to second- (*M*_2_) and third-order (*M*_3_) motion compensation are asymmetric in time and shape as shown in Figure 1. Our waveforms are not constrained a priori to a specific shape (i.e., trapezoidal, sinusoidal, etc.) since limiting the shape of the waveform makes the design suboptimal (see Figure S7). The waveforms in Figure 1 do not reach *G*_max_ in the left side of the waveform, due to the Maxwell-compensation constraint. If we release this constraint, the waveform gets closer to the *G*_max_ on either side of the refocusing pulse. Since the NOW toolbox does not guarantee a global minimum the optimization is repeated 10 times with random waveforms for initialization (Figure S9) and the result with the highest *b*-value is selected (see References 26 and 27 for more details). The NOW toolbox provides the waveforms as text files that can be read by the sequence. The sequence interpolates the gradient waveforms linearly to the raster time that is used by the scanner. This interpolated waveform is used to calculate the *b*-value. A potentially remaining zeroth-order moment, due to the interpolation, is compensated by adding a short, small balancing gradient pulse. The optimization processing time was on the order of minutes.^26^

### 2.3 Data acquisition

Routine GRE and TRUEFISP sequences were used for cardiac planning and cine-imaging, whereas cardiac DWI was performed with a prototype pulse sequence that enabled diffusion encoding with user-defined gradient waveforms and with EPI readout.^28^ The cine images were acquired in short-axis orientation for apical, mid, and basal slices. DWI was performed at the same location and orientation as the cine imaging. The phase encoding direction was systematically varied in scout DW images (step size of 30°) and the phase encoding orientation providing the best image quality was chosen for the full cardiac DWI acquisition in each subject. The cardiac DWI parameters were: TR = 3RR-intervals, field-of-view = 320 × 195 mm^2^, in-plane resolution = 2.3 × 2.3 mm^2^, slice thickness = 8 mm, three short axis slices (base, mid, and apical), partial Fourier factor = 7/8, no parallel imaging, bandwidth = 2012 Hz/pixel and local subject-specific shimming. Each full data set comprised of *b* = 100, 450, and 1000 s/mm^2^ in 3, 30, and 30 directions with 12, 6, and 6 repetitions, respectively, for both *M*_2_ and *M*_3_. Data were acquired with ECG-gating and under free-breathing.^8^ Neither respiratory navigation nor respiratory gating were used. Navigators can pro-long the acquisition time significantly as they depend on the navigator efficiency. It is common practice to exclude motion-corrupted diffusion-weighted images in post-processing prior to diffusion tensor fitting given that the image space is oversampled with respect to number of diffusion encoding directions and repetitions. Importantly, we previously did not find any difference in diffusion biomarkers in using this approach versus data acquired with respiratory navigator (unpublished data). The latter approach came at the expense of significantly prolonged acquisition times. Saturation bands were placed around the heart to suppress the signal from outside the volume of interest. Fat suppression was performed using the SPAIR method.^29^ The trigger delay was defined as ∼20% of end-systole as determined from the cine images to acquire the images at peak systole, that is, maximal wall thickness. The total acquisition time was around one hour. Both magnitude and phase data were collected and used to generate complex-valued images. The data associated with this paper are available from the University of Leeds Data Repository, https://doi.org/10.5518/1511.

The maximum gradient strength used in this study for acceleration-compensation acquisition (*M*_2_) to generate the *b*-value of 1000 s/mm^2^ was 296.4 mT/m and the maximum slew-rate was 80 T/m/s which resulted in an TE of 74 ms. For the third-order motion compensation (*M*_3_), the maximum gradient strength was 293.2 mT/m with a maximum slew-rate of 79.2 T/m/s to provide the *b*-value of 1000 s/mm^2^ with the TE of 80 ms.

Separate noise-only data sets (magnitude and phase) were acquired using the same sequence without RF pulses^30^ and with a TR of 730 ms.

### 2.4 Data analysis

The phase variation in each complex-valued diffusion-weighted image was removed using the method proposed by Eichner et al.^31^ An in-house developed toolbox was used for further postprocessing.^32–34^ Real-valued diffusion-weighted images were first registered: for each slice, all low *b*-value images were registered to one user-specified low *b*-value image, and then all images were registered to the mean of the co-registered low *b*-value images. The two-dimensional registration was performed with SimpleElastix,^33^ with rigid transformation, separately for basal, middle, and apical slices. Next, an outlier rejection technique was used to remove the outliers (e.g., the images with misregistration or motion corruption) from the data.^32^ Last, the diffusion tensor was fitted using weighted linear least squares regression^35^ to the data, and diffusion metrics such as fractional anisotropy (FA), mean diffusivity (MD), helix angle (HA), and secondary eigenvector angle (E2A)^36^ were extracted for each voxel.^32^ The left ventricle in each slice was segmented manually using an in-house developed toolbox.^32^ Parts of the left ventricle corrupted by susceptibility-related distortion were not included in the averaging for the global metrics. The noise level, *σ*, was measured as the SD of the real part of the noise data (acquired without RF pulses) in the image domain from 256 repetitions. The SNR of the data is defined as SNR = *S*/*σ*, where *S* is the measured signal intensity for each *b*-value and direction.^14^ The data were divided into four sets to conduct the experiments:

- *M*_2_ with *b* = 100 and 450 s/mm^2^
- *M*_2_ with *b* = 100 and 1000 s/mm^2^
- *M*_3_ with *b* = 100 and 450 s/mm^2^
- *M*_3_ with *b* = 100 and 1000 s/mm^2^

Bland−Altman plots were used to compare the diffusion metrics (FA, MD, and E2A) obtained from *M*_2_ versus *M*_3_ as well as *b*_max_ = 450 s/mm^2^ versus *b*_max_ = 1000 s/mm^2^. The mean value of MD, FA, and E2A in each slice was used for comparison. To determine the statistical significance between different schemes, the Wilcoxon signed-rank test was used where a *p*-value less than or equal to 0.05 was considered statistically significant.

## 3 Results

**Figure 2:**
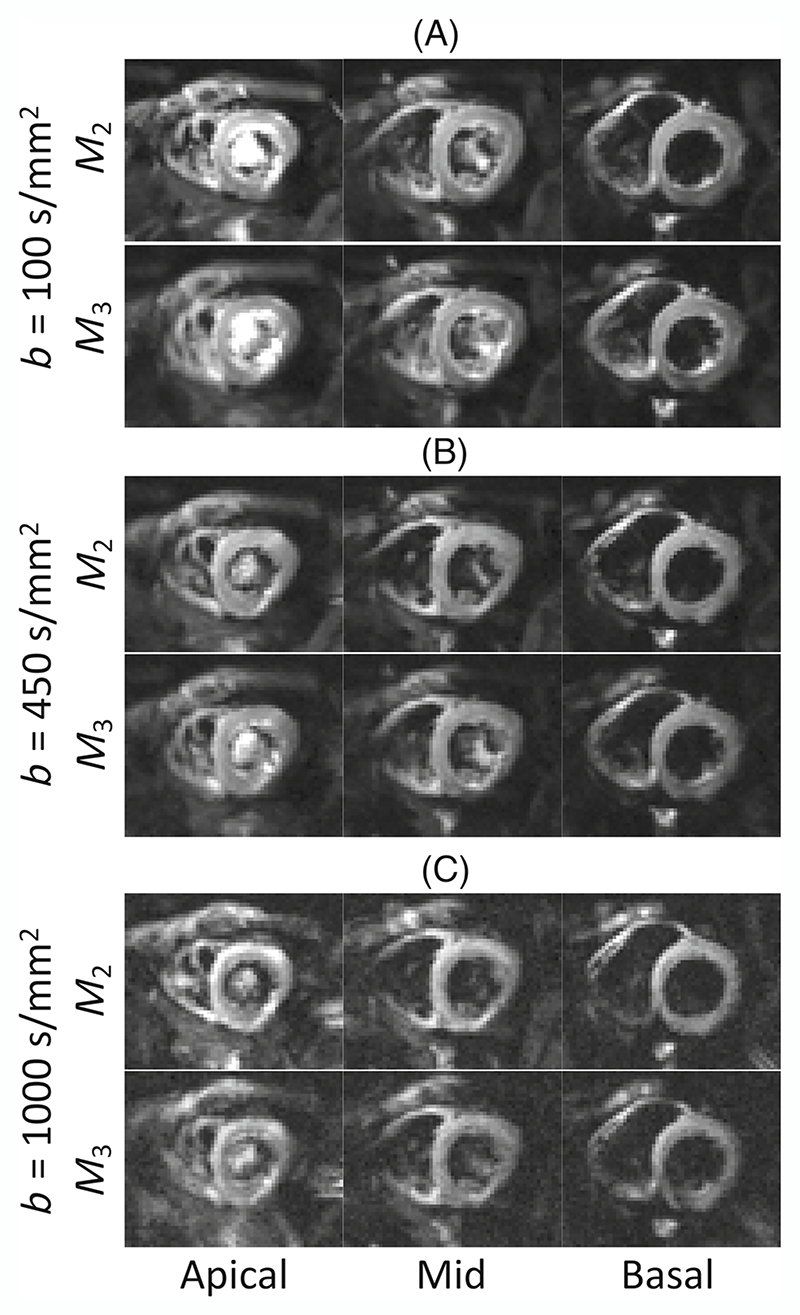
Representative cardiac diffusion-weighted images averaged over six repeats of a single diffusion direction acquired in basal, mid and apical slices with *b* = 100, 450, and 1000 s/mm^2^ (panels A–C) using second (*M*_2_, TE = 74 ms) and third-order motion compensation (*M*_3_, TE = 80 ms). *M*_2_- and *M*_3_-compensated images for each *b*-value are shown with the same window/grayscale level.

**Figure 3:**
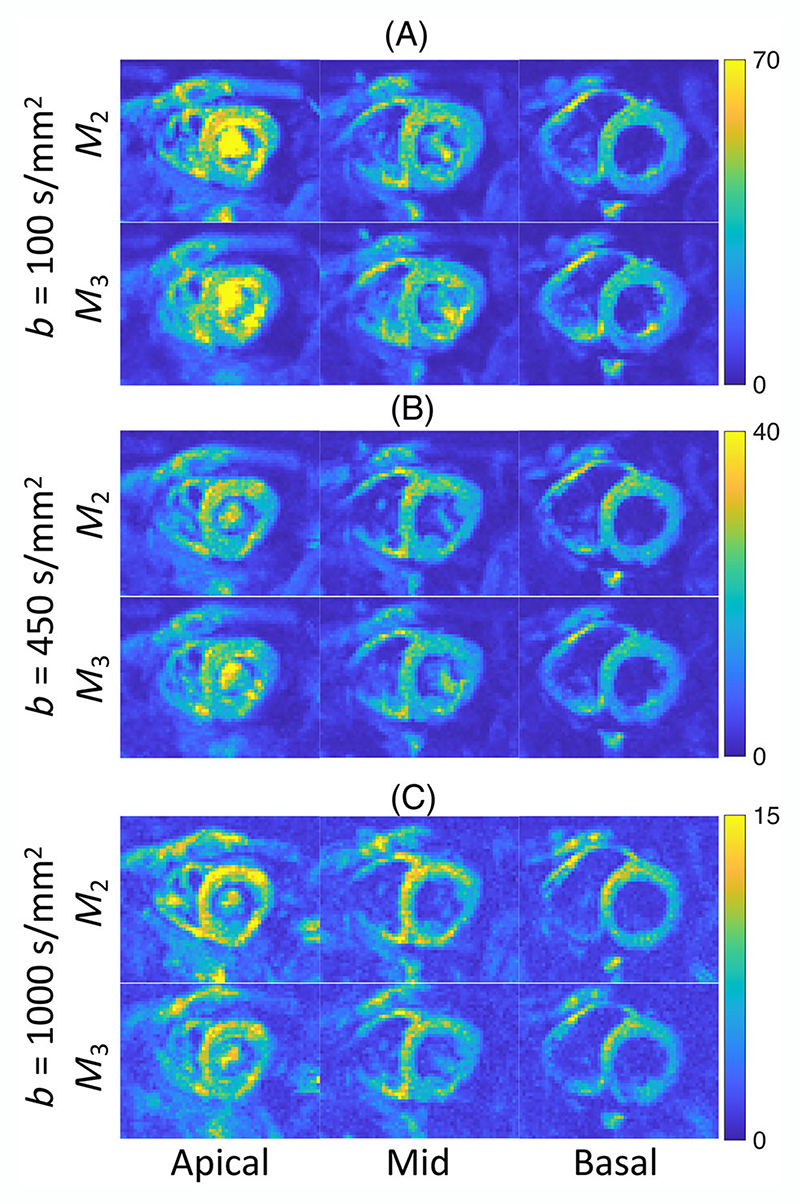
Signal-to-noise-ratio (SNR) maps obtained in basal, mid, and apical slices from a single diffusion direction (the same diffusion direction as Figure 2) with *b* = 100, 450, and 1000 s/mm^2^ (panels A–C, respectively) using second (*M*_2_, TE = 74 ms) and third-order motion compensation (*M*_3_, TE = 80 ms).

Figure 2 shows representative diffusion-weighted images averaged over six repeats of a single diffusion direction acquired with *b* = 100, 450, and 1000 s/mm^2^ using second- and third-order motion compensation (*M*_2_ and *M*_3_). 2% of diffusion-weighted images were discarded due to poor image quality and signal dropout and on average 18% of the voxels were excluded per data set for calculating the mean global metrics due to the susceptibility-related distortions. An example diffusion weighted image for each subject is shown in Figure S8. The measured SNR of the *M*_2_- and *M*_3_- compensated images at *b* = 100 s/mm^2^ were 33 ± 11, and 29 ± 10, respectively, over the left ventricle (Figure 3). This decrease in SNR is in line with the increase in TE from *M*_2_ (TE = 74 ms) to *M*_3_ (TE = 80 ms), assuming a T2 of 46 ms^37^ $\left(\exp(-(TE_{M_{3}}-{\text{TE}}_{M_{2}})/\text{T}2)=\exp(-6/46)∼0.88\approx 29/33\right)$. While the SNR decreases slightly going from *b* = 100 to 450 s/mm^2^, the SNR change is more pronounced from *b* = 450 to 1000 s/mm^2^ (Figure 3). Each *b*-value uses a different window/level for better visibility.

**Figure 4:**
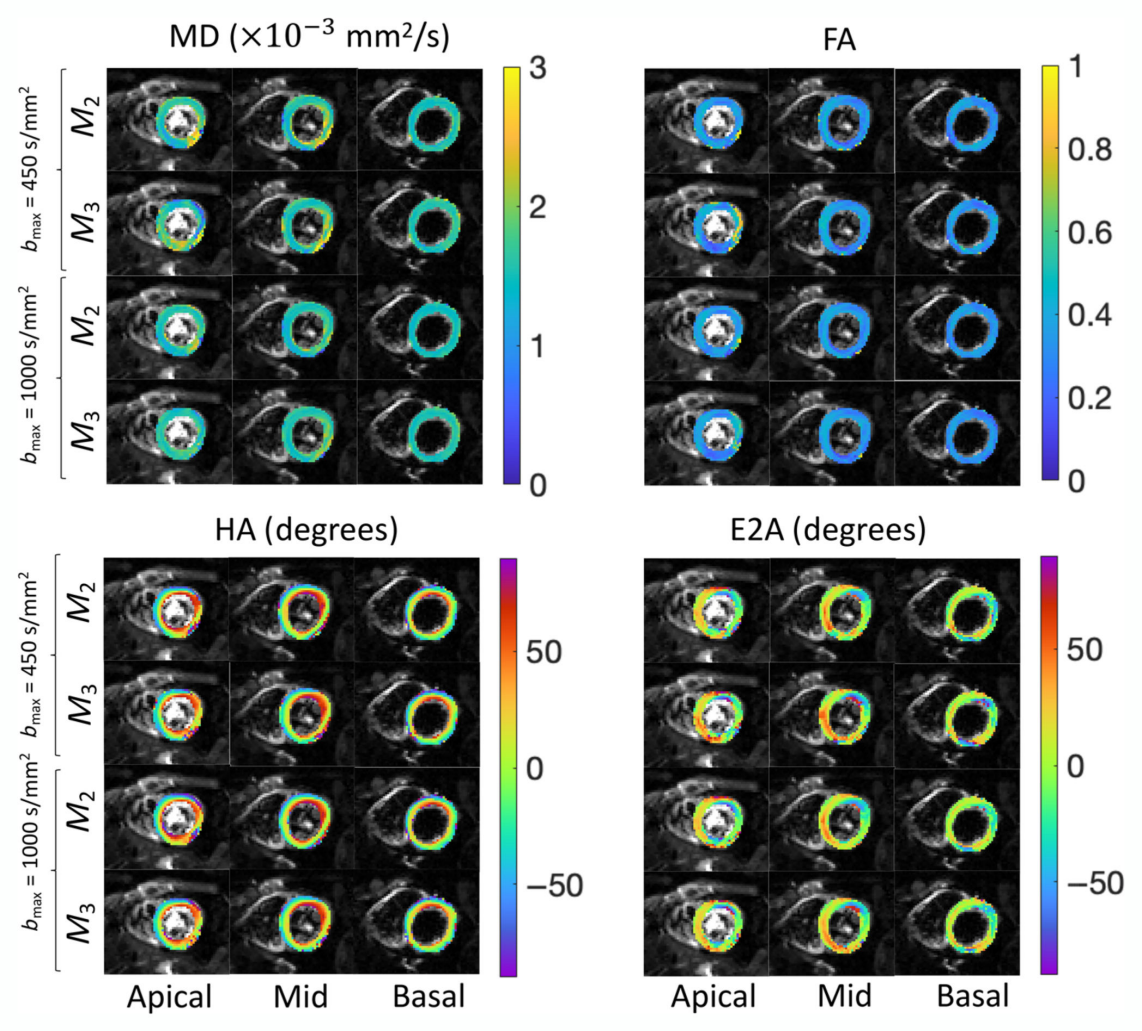
Examples of estimated mean diffusivity (MD), fractional anisotropy (FA), helix angle (HA), and secondary eigenvector angle (E2A) from data acquired using second- and third-order motion compensated waveform (*M*_2_ and *M*_3_) with *b*_max_ = 450 s/mm^2^, *b*_max_ = 1000 s/mm^2^ for basal, mid, and apical slices.

**Figure 5:**
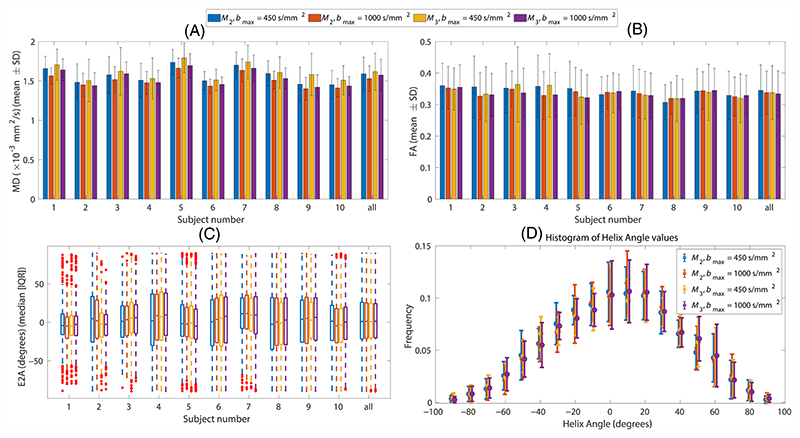
Mean and SD of (A) mean diffusivity (MD), (B) fractional anisotropy (FA), (C) median and interquartile range (IQR) for secondary eigenvector angle (E2A), and (D) histogram of helix angles (HA) over left ventricular mask. Each color shows one of the schemes. The bars shown as “all” represent the mean and SD over all 10 subjects. The red crosses in (C) show the outliers.

Figure 4 shows the MD, FA, E2A, and HA maps from data acquired using second- and third-order motion compensated waveforms (*M*_2_ and *M*_3_) with *b*_max_ = 450 s/mm^2^ and *b*_max_ = 1000 s/mm^2^ for three different slices. Helix angle maps obtained from acceleration-compensated diffusion encoding demonstrate the distinctive rotation from positive to negative helix angles from the subendocardium and subepicardium.^38^ The transmural rotation of the helix angle is also apparent in the helix angle maps derived from the *M*_3_-compensated diffusion encoding. The mean and SD of MD, FA, and E2A for all 10 subjects are shown in Figure 5A−C. Histogram of HA values for all four schemes is shown in Figure 5D. The histograms of HA are similar for all four scenarios. The MD values are higher for *b*_max_ = 450 s/mm^2^ compared to *b*_max_ = 1000 s/mm^2^ for both motion compensation schemes (Table 1). The MD values are consistently higher in *M*_3_ compared to *M*_2_ for both *b*_max_ values (Table 1). There is no specific trend in FA, E2A, and HA values between different schemes.

**Figure 6:**
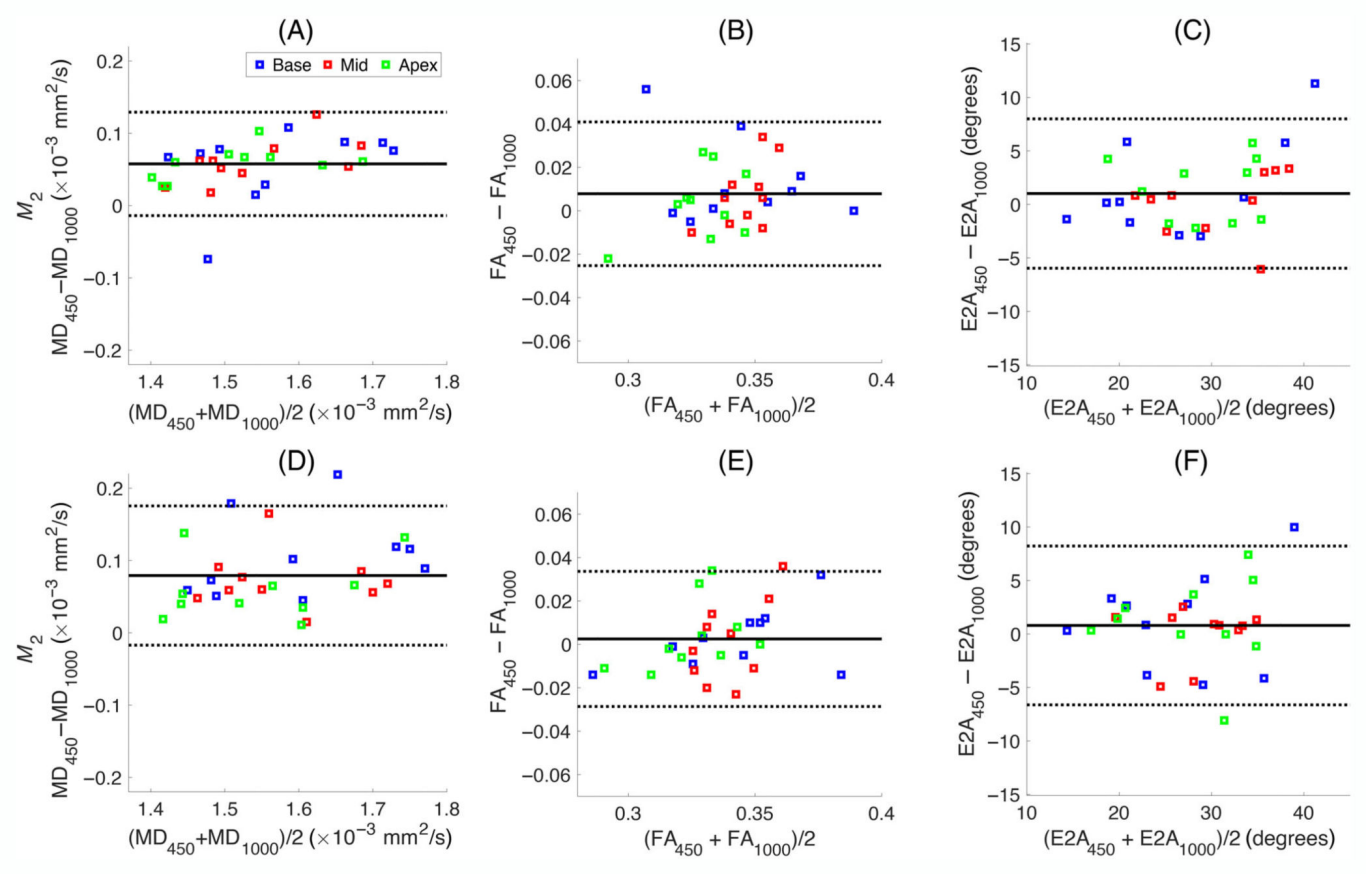
Bland−Altman plots, comparing the mean diffusivity (MD), fractional anisotropy (FA), and secondary eigenvector angle (E2A) estimated using *b*_max_ = 450 s/mm^2^, and *b*_max_ = 1000 s/mm^2^ for second- (A−C) and third-order motion compensation (D−F) (*M*_2_ and *M*_3_). Mean difference ± 1.96 SD is given by solid and dashed black lines, respectively (*N* = 10 subjects). The results for basal, middle, and apical slices are shown by blue, red, and green squares, respectively. The subscripts indicate the *b*_max_ that was used to estimate the metric.

**Figure 7:**
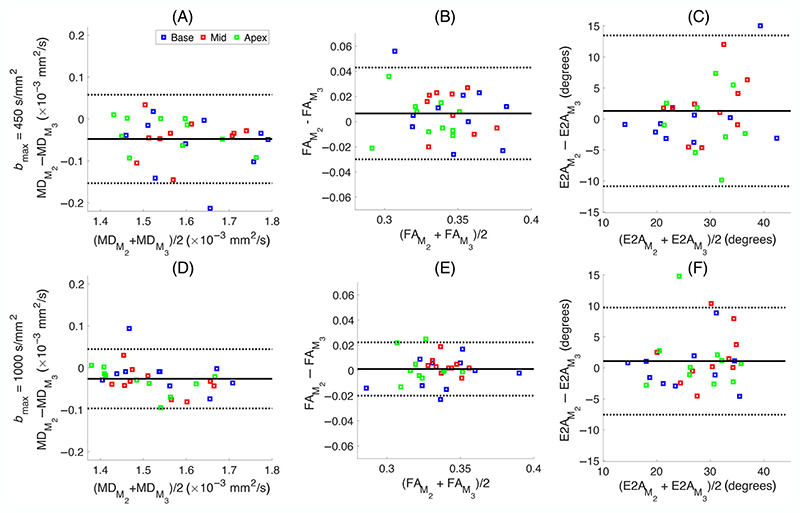
Bland–Altman plots, comparing the mean diffusivity (MD), fractional anisotropy (FA), and secondary eigenvector angle (E2A) estimated using second- and third-order motion compensation (*M*_2_ and *M*_3_) for *b*_max_ = 450 s/mm^2^, (A−C) and *b*_max_ = 1000 s/mm^2^ (D−F). Mean difference ± 1.96 SD is given by solid and dashed black lines, respectively (*N* = 10 subjects). The results for basal, middle, and apical slices are shown by blue, red, and green squares, respectively. The subscript “*M*_2_” shows the metric was obtained using second-order motion compensation and the ones with subscript “*M*_3_” are estimated using third-order motion compensation.

To investigate the difference between diffusion metrics from different schemes further, we performed a Bland–Altman analysis, shown in Figures 6 and 7, for MD, FA, and E2A values obtained with different experimental settings. Figure 6 depicts the effect of different *b*-values, while Figure 7 illustrates the impact of the order of motion-compensation. The top row of Figure 6 shows the comparison between (a) MD, (b) FA, (c) E2A values obtained using *b*_max_ = 450 s/mm^2^ (MD_450_, FA_450_, and E2A_450_) and the ones estimated using *b*_max_ = 1000 s/mm^2^ (MD_1000_, FA_1000_, and E2A_1000_) for the second-order motion compensation. The second row (D−F) shows the results for the corresponding comparison between *b*_max_ = 450 s/mm^2^ and *b*_max_ = 1000 s/mm^2^ for the third-order motion compensation (*M*_3_) scheme, respectively. There is a statistically significant difference of (0.06 ± 0.04)× 10^−3^ mm^2^/s (*p* = 1.6*e* − 9) between MD_450_ and MD_1000_ for *M*_2_ scenario, while this difference is slightly higher for the *M*_3_ case, (0.08 ± 0.05)× 10^−3^ mm^2^/s (*p* = 1*e* − 9), (Table 2). The mean difference between FA and E2A from the two *b*_max_ is almost negligible (Figure 6B,C,E,F). Figure 7 shows the comparison between the MD, FA, and E2A values using second- and third-order motion compensation (*M*_2_ and *M*_3_) for *b*_max_ = 450 s/mm^2^ and *b*_max_ = 1000 s/mm^2^. The difference between ${\text{MD}}_{M_{2}}$ and ${\text{MD}}_{M3}$ for *b*_max_ = 450 s/mm^2^ was (−0.05 ± 0.05)× 10^−3^ mm^2^/s (*p* = 4*e* − 5) which is slightly higher than the difference using *b*_max_ = 1000 s/mm^2^, −0.03 ± 0.0.03 × 10^−3^ mm^2^/s (*p* = 4*e* − 4) (Table 2). The difference between FA and E2A obtained using *M*_2_ and *M*_3_ is almost negligible (Figure 7B,C,E,F).

## 4 Discussion

This work demonstrates the feasibility and benefits of using gradients that are much stronger than those commonly available in the clinical routine setting, for cardiac diffusion MRI applications. Conventional cardiac imaging sequences (such as cine-MRI) could be applied without any further adjustments. Using the Connectom MR system, we can reach a *b*-value of 1000 s/mm^2^ with a minimum TE = 74 ms for the given imaging parameters and optimized waveforms. Notably, the same *b*-value on clinical routine systems with *G*_max_ = 80 mT/m would need a TE of at least 100 ms. The approximately 25 ms shorter TE improves the SNR nearly twofold due to the short T2 of cardiac tissue: assuming a T2 of around 46 ms as reported in the literature^37^ the SNR increase is exp(−74/46)/ exp(−100/46) ≈ 1.76. It can be expected that modern hard- and software (such as parallel transmit) will provide further TE savings but this was beyond the current capabilities of the Connectom MR scanner. The high-gradient amplitudes and relatively thick slices made it essential to include Maxwell compensation at only a small TE increase of 4 ms compared to the uncompensated case (see also Figure S7).

### 4.1 Effect of motion compensation order (M_2_ vs. M_3_)

Utilizing the high-performance gradient system of the Connectom MR scanner, we were able to demonstrate third-order motion-compensated cardiac diffusion MRI with a TE of 80 ms for *b*_max_ = 1000 s/mm^2^ which is similar to the TE routinely used in clinical scanners for the *M*_2_-compensated SE-sequences at *b*_max_ = 450 s/mm^2^.^9,10,8^

Previous studies demonstrated that SE (diffusion MRI) with acceleration compensated diffusion gradients (*M*_2_) provided both sufficient SNR and insensitivity to motion (in at least the systolic phase)^3,4,7,6^ to get satisfactory cardiac diffusion images in vivo. Our work confirms this finding. However, considering that the participants in this study were healthy volunteers, with regular heartbeats, the quality of the images acquired using *M*_2_ and *M*_3_ were not qualitatively different. The third-order motion compensation scheme (*M*_3_) may result in better quality images in patients with a less regular heartbeat.

The slight difference between MD values using *M*_2_ and *M*_3_ could be due to the difference in the shape and timing of the diffusion gradient waveforms (Figure 1): the presence of time-dependent diffusion processes^39^ can affect MD value estimation from second- and third-order motion compensated (*M*_2_ and *M*_3_) acquisitions. FA and the angular diffusion metrics (E2A and HA) were found to be the same for both motion compensation orders.

### 4.2 Effect of *b*_max_ (450 vs. 1000 s/mm^2^)

The MD and FA values using *b*_max_ = 450 s/mm^2^ and *b*_max_ = 1000 s/mm^2^ are in agreement with the results reported for cardiac DWI spin echo sequences.^40,41,10,4,6^ A reduction in MD value is observed when the *b*-value is increased from *b*_max_ = 450 s/mm^2^ to *b*_max_ = 1000 s/mm^2^. This reduction is statistically significant and can be attributed to non-Gaussian diffusion effects which become more pronounced at higher *b*-values.^15^ FA and HA values obtained from both *b*_max_ were similar. E2A derived from both *b*-value data was comparable, albeit at the lower end of the range reported in the literature for SE in systole. This could be due to the timing of the DWI acquisition in the cardiac cycle. We aimed the acquisition to be in the end-systolic phase but if the timing (trigger delay) is slightly off, the data may be acquired closer to the mid-systolic phase which would result in lower E2A values.^42^

### 4.3 Limitations and future work

The diffusion-weighted technique used in our study was based on a SE EPI sequence. It is well recognized that EPI with long readouts is prone to geometrical distortions and intensity variations: off-resonance effects result in a reduced/increased encoding bandwidth in the phase-encoding direction.^43^ These off-resonances can be caused by susceptibility-induced local gradients^44^ between myocardium, deoxygenated blood, and air, which are particularly pronounced around the posterior vein.^45,46^ For the highest possible SNR, the centre of k-space should coincide with the SE condition. Since EPI does typically not follow a center-out trajectory, it prolongs TE. In addition, the minimal TE becomes dependent on imaging parameters, such as field of view, resolution, and readout bandwidth. The lack of two-dimensional RF pulses (readily available on scanners with newer software and state-of-the-art hardware) necessitated the acquisition of a larger field of view in phase encoding direction, which increased TE further. A larger field of view in turn was necessary to avoid aliasing artifacts from signals not fully suppressed by the saturation bands. Future work will see the implementation of short, optimized multidimensional RF pulses as proposed by Vinding et al.^47^ to overcome this limitation. To minimize the risk of peripheral nervous system, cardiac stimulation, and the occurrence of magnetophosphenes generated by the diffusion encoding waveforms, we compromised on the slew rate while capitalizing on the maximum gradient strength.^48^ This is based on our simulations (designed waveforms in Figures S4 and S5) demonstrating that combining maximum gradient strength with the associated lower slew rate results in the shortest TE (see Figures S4 and S5, for more details). Thus, the waveforms here used a slew rate of ∼ 80 T/m/s instead of 200 T/m/s theoretically possible. This added 16/14 ms to the TE of the *M*_2_/*M*_3_ acquisitions, respectively. Notably, using *M*_1_ reduces the TE by 4−70 ms. We therefore opted to maintain the higher level of motion compensation.

The TE can in principle also be shortened through various complementary approaches: (1) parallel imaging techniques^49,50^ can reduce the time to the k-space center, but result in an SNR penalty. (2) inner volume imaging^51^ with the 90° and 180° RF pulses applied orthogonal to each other allows for a smaller FOV in phase-encoding direction, and in turn can translate into a shorter readout duration at the same spatial resolution. We refrained from implementing this technique as this will impact the three-slice acquisition protocol performed in this study. The effective TR for each slice was 3 RR intervals (i.e., approximately 3 s). Thus, acquiring the same number of slices would have resulted in threefold longer scan time (at the same T1 saturation) or caused significantly higher T1 saturation (i.e., lower SNR) at the same scan time. (3) Replacing the EPI with a spiral readout has been demonstrated to improve SNR on high-amplitude gradient systems^18,52,53^ and in cardiac DWI^54–57^ and is expected to be particularly beneficial in this context.

Reaching a *b*-value of 1000 s/mm^2^ in a reasonable TE is an important achievement and opens the field for further investigations. Future work will explore non-Gaussian diffusion such as diffusion kurtosis imaging in more detail.

In this work, we only acquired the data in the systolic phase of the cardiac cycle. The main reason is that this study aimed to show the feasibility of cardiac diffusion MRI using strong gradients and spin echo based acquisitions. In addition, most of the previous spin echo based works in the literature are focused on the cardiac diffusion MRI in the systolic phase since diastolic acquisitions are challenging using SE-based sequences.^58^ To make comparisons straightforward we focused on the systolic images, but our future work will consider the cardiac diffusion MRI in the diastolic phase.

## 5 Conclusion

We successfully demonstrated SE-based cardiac DWI acquisitions of the human heart using a Connectom scanner, which has not been performed to date. The high-performance gradients on such a scanner, which are about 4−8× more powerful than those available on clinical routine MR systems, enabled us to acquire cardiac diffusion-weighted images with third-order motion compensation (i.e., compensating for velocity, acceleration, and jerk) at a maximum *b*-value of 1000 s/mm^2^ while achieving TEs comparable to second-order motion compensated diffusion gradients at *b* = 450 s/mm^2^. We observed a statistically significant reduction in MD values obtained using *b* = 1000 s/mm^2^ compared to *b* = 450 s/mm^2^. This can be due to the non-Gaussian diffusion which is more pronounced at higher *b*-values and opens a new avenue for microstructural investigation of the cardiac tissue. Future work will also establish whether or not *M*_3_ motion compensation will be sufficient to improve cardiac DWI SE acquisitions in diastole.

## Supplementary Material

Supporting Information

Supporting Information Legand

## Figures and Tables

**Table 1:** The mean and SD of mean diffusivity (MD), fractional anisotropy (FA), and median (interquartile range [25%−75%]) secondary eigenvector angle (E2A) values inside a left ventricle mask over all three slices and over all 10 subjects.

*b*_max_ (s/mm^2^)		MD (×10^−3^ mm^2^/s) (mean ± SD)	FA (mean ± SD)	E2A (degrees) (median [IQR])
450	*M*_2_	1.59 ± 0.21	0.34 ± 0.08	1 (−22 26)
	*M*_3_	1.62 ± 0.23	0.34 ± 0.09	1 (−21 25)
1000	*M*_2_	1.53 ± 0.16	0.34 ± 0.07	1 (−20 24)
	*M*_3_	1.57 ± 0.20	0.33 ± 0.07	1 (−20 24)

**Table 2:** Mean difference ± SD of mean diffusivity (MD), fractional anisotropy (FA), and secondary eigenvector angle |E2A| inside a left ventricle mask for different schemes.

	*b*_max_ = 450 versus 1000 (s/mm^2^), *M*_2_	*b*_max_ = 450 versus 1000 (s/mm^2^), *M*_3_
ΔMD(×10^−3^ mm^2^/s)	0.06 ± 0.04 (*p* = 1.6e−9) (Figure 6A)	0.08 ± 0.05 (*p* = 1e−9) (Figure 6D)
ΔMD percentage	4%	5%
ΔFA	0.008 ± 0.016 (*p* = 0.02) (Figure 6B)	0.002 ± 0.016 (*p* = 0.4) (Figure 6E)
ΔFA percentage	2%	0.6%
Δ|E2A|(degrees)	1 ± 3 (*p* = 0.13) (Figure 6C)	1 ± 4 (*p* = 0.26) (Figure 6F)
Δ|E2A| percentage	4%	4%
	*M*_2_ versus *M*_3_, *b*_max_ = 450 (s/mm^2^)	*M*_2_ versus *M*_3_, *b*_max_ = 1000 (s/mm^2^)
ΔMD (×10^−3^ mm^2^/s)	−0.05 ± 0.05 (*p* = 4e−5) (Figure 7A)	−0.03 ± 0.03 (*p* = 4e−4) (Figure 7D)
ΔMD percentage	−3%	−2%
ΔFA	0.006 ± 0.018 (*p* = 0.06) (Figure 7B)	0.001 ± 0.011 (*p* = 0.55) (Figure 7E)
ΔFA percentage	2%	0.3%
Δ|E2A|(degrees)	1 ± 6 (*p* = 0.26) (Figure 7C)	1 ± 4 (*p* = 0.19) (Figure 7F)
Δ|E2A| percentage	4%	4%
