# Supplementary material for: In vivo diffusion MRI of the human heart using a 300 mT/m gradient system
Source: Magn Reson Med. Author manuscript; Available in PMC 2025 Mar 13. (PMC7617480; doi:10.1002/mrm.30118)
Supplement: Supporting Information [file EMS203648-supplement-Supporting_Information.pdf]

Supporting information

Magnetophosphenes

Physiological effects (including the peripheral nerve stimulation (PNS)) prohibit the combination of both maximum gradient strength and maximum slew rate. Real-time monitors built into the MR scanner architecture ensure that both the defined PNS threshold Setsompop et al. (2013) and, more importantly, the cardiac stimulation threshold (based on International Electrotechnical Commission (IEC) guidelines (Commission et al., 2002; CODE and PRIX, 2015))) are not exceeded. Nevertheless, PNS can still be induced and therefore needs to be investigated (and minimized) for any new applications Molendowska et al. (2021).

In addition to the PNS and cardiac stimulation limits, a third effect concerns magnetophosphenes. Phosphene induction, in general, is a visual sensation of light not caused by external visual stimuli, but by mechanical and / or electric stimuli on the retina (Oster, 1970). Specifically, magnetophosphenes are caused by a magnetically induced electric field (Marg, 1991; Saunders and Jefferys, 2007; Laakso and Hirata, 2012). This can be caused by the person moving in the static magnetic field of MR scanners (Weintraub et al., 2007) or by switching magnetic field gradients with amplitudes larger than 130 mT/m (Setsompop et al., 2013), although no detailed threshold study has been performed so far. Notably, magnetophosphenes are not considered to represent an adverse health concern Marg (1991); Schutter and Hortensius (2010); Taylor et al. (2010). While confirmed by the International Commission on Non-Ionizing Radiation Protection (ICNIRP) guidelines for occupational exposure their production should nevertheless be avoided on Non-Ionizing Radiation Protection et al. (2010). In MRI, patient positions where a subject's head is located away from the isocentre, e.g. as for cardiac MRI, are more prone to cause magnetophosphenes Setsompop et al. (2013); Molendowska et al. (2022). Thus, this phenomenon needs to be addressed for novel applications outside the brain.

A previous study investigated physiological effects of human body imaging with 300 mT/m gradients when applying a continuous train of 128 trapezoidal bipolar gradient pulses (Molendowska et al., 2022). Here, we extended the investigations for the heart in the isocentre using motion-compensated diffusion gradient waveforms. The diffusion gradient waveforms were designed asymmetric in time and shape. Motion compensation up to order 3 (i.e. M_1, M_2, M_3) was used. In addition, an acquisition without motion compensation (i.e. trapezoidal diffusion gradient – M_0) served as reference (Figure S1). To separate the effect of diffusion gradient waveform from the readout gradients, the experiment was repeated for a setup with no readout.

Ten healthy participants (age range 29-49 years old (35.2 ± 7.3), weight range of 47-90 kg (67.7 ± 14.31 kg), six females and four males) were recruited to study the possible existence of magnetophosphenes in the cardiac position. There were no images collected as no RF pulses were applied. Waveforms and readouts were applied in random order within and between participants. After each protocol, the participant was asked to provide feedback about the perception of magnetophosphenes. Other sequence parameters were: TR = 3 s, bandwidth = 1494 Hz/pixel, b = $1000\,\mathrm{s/mm^2}$ in X, Y, Z, and orthogonal (XYZ) directions. Despite the higher nominal maximum slew rate available, slew rates were reduced to avoid exceeding scanner-defined peripheral nerve stimulation (PNS) and cardiac stimulation limits (Ham et al., 1997). The total investigation time was one hour. The occurrence of the magnetophosphenes effect was counted among the participants and a percentage was provided for each motion compensation order (Figure S2). The plots do not show any clear trend in changes in the magnetophosphenes perception by increasing the motion compensation order from one to three (M_1 to M_3). Also, no clear trend between EPI and no readout can be observed. As shown in the figure, the percentage of magnetophosphenes perception by the participants is less than 20% which was distributed amongst all individuals.

Details on the gradient waveform design

The gradient coil was tested for PNS at the factory using a standard threshold detection protocol with a train of trapezoidal gradient pulses Setsompop et al. (2013). Figure S3 shows the stimulation curves (provided by vendor) for G_y (anterior-posterior in patient coordinate system) which is reported (Molendowska et al., 2022) as the most limiting axis for the PNS threshold. The hardware limit (G_{max} vs ramp

time) is $G_{max} = 300\,mT/m$ with slew rate of 200 T/m/s (black line), while this combination cannot be used due to the PNS and cardiac stimulation limits (blue and red lines). For $G_{max} = 300\,mT/m$ the maximum slew-rate allowed by the system is around 90 T/m/s, this limit is nearly the same even for G_{max} of $200\,mT/m$. Note that Ramp time here refers to 1/ slew rate.
Table S1 shows the minimum echo time for each combination of G_{max} and maximum slew-rate (S_{max}). As it is shown by reducing the G_{max} the echo time is increased. Note that the G_{max} and S_{max} reported in this table are based on the train of trapezoidal gradient pulses, these limits might be slightly different when other gradient waveforms such as the ones in this paper are used. This explains why our study did not use the $G_{max} = 300mT$ with the slew rate of 89.8 T/m/s.
Figure S4 and S5 show the gradient waveforms optimized for different G_{max} and S_{max} (Table S1). As both figures show, by reducing the G_{max}, a longer time is needed to play out the gradient and provide the maximum b-value since we are limited by the S_{max}.
The amount of predicted PNS using the SAFE model Hebrank et al. (2000) (`https://github.com/filip-szczepankiewicz/safe_pns_prediction`) for the waveforms used in this study is provided in Figure S6 which is 81% and 78% in Y direction for M_2 and M_3-compensated waveforms respectively. Note that the Y direction (anterior-posterior in patient coordinate system) is the most limiting axis for the PNS threshold. As explained in the method section, the waveforms in this study are asymmetric and Maxwell-compensated. Enforcing symmetry on the waveforms (Figure S7 , top left), increases the echo time by 3 ms (77 instead of 74 ms in our design). Removing the Maxwell compensation constraint (Figure S7 , top right) reduces the echo time to 70 ms (saving 4 ms at the expense of accepting concomitant field effects Szczepankiewicz et al. (2019)). Enforcing a trapezoidal shape (Figure S7 , bottom left) on the waveform adds 4 ms to the echo time (78 ms). Finally, reducing the motion compensation order to M_1 (Figure S7 , bottom right) saves only 4 ms at the expense of accepting the non-compensated acceleration effect.

Table S1 : The maximum slew-rate (S_{max}) allowed by the system for each G_{max} (provided by the vendor, Figure S3) with the corresponding minimum echo time for second and third order motion compensation (M_2 and M_3).

G_{max} [mT/m]	20.9	27.6	35.8	62.5	104.4	144.7	190.9	228.2	300
S_{max} [T/m/s]	200	200	179	125	104.4	96.5	92.7	91.3	89.8
TE [ms] (M_2)	179	155	138	105	87	80	75	75	73
TE [ms] (M_3)	228	198	168	128	99	89	86	80	79

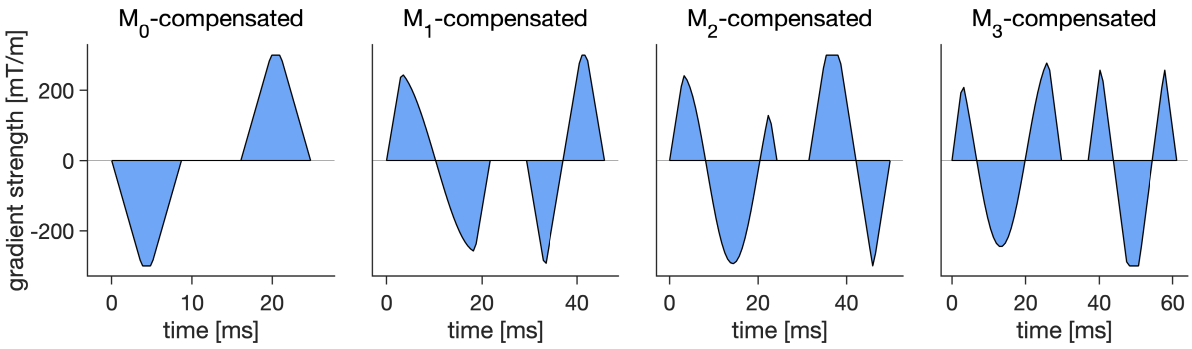

Figure S1 : Optimized motion compensated waveforms up to order three (M_0, M_1, M_2, M_3). The waveforms were numerically optimized with the NOW toolbox to provide the shortest echo time for b = 1000 s/mm^2, $G_{max} = 300\,mT/m$, and a maximum slew rate of $80\,T/m/s$.

References

CODE, P., PRIX, C.. Medical electrical equipment–part 2-33: Particular requirements for the safety of magnetic resonance equipment for medical diagnosis appareils électromédicaux–partie 2-33: Règles particulières de sécurité relatives aux appareils à résonance 2015;.

Commission, I.E., et al. Medical electrical equipment. particular requirements for the safety of magnetic resonance equipment for medical diagnosis. International Standard, IEC60601-2-33 2002;.

Ham, C., Engels, J., Van de Wiel, G., Machielsen, A.. Peripheral nerve stimulation during MRI: effects of high gradient amplitudes and switching rates. Journal of Magnetic Resonance Imaging 1997;7(5):933–937.

Hebrank, F.X., Gebhardt, M., et al. SAFE-model-a new method for predicting peripheral nerve stimulations in MRI. In: Proc Intl Soc Mag Res Med. 2000. p. 2007.

Laakso, I., Hirata, A.. Computational analysis of thresholds for magnetophosphenes. Physics in Medicine & Biology 2012;57(19):6147.

Marg, E.. Magnetostimulation of vision: direct noninvasive stimulation of the retina and the visual brain. Optometry and vision science: official publication of the American Academy of Optometry 1991;68(6):427–440.

Molendowska, M., Fasano, F., Rudrapatna, U., Kimmlingen, R., Jones, D., Kusmia, S., Tax, C., Evans, J.. Going below the neck: Physiological limits on use of 300 mT/m gradients in the human body. In: Proceedings of the 29th Annual Meeting of ISMRM. 2021. .

Molendowska, M., Fasano, F., Rudrapatna, U., Kimmlingen, R., Jones, D.K., Kusmia, S., Tax, C.M., Evans, C.J.. Physiological effects of human body imaging with 300 mt/m gradients. Magnetic Resonance in Medicine 2022;87(5):2512–2520.

on Non-Ionizing Radiation Protection, I.C., et al. Guidelines for limiting exposure to time-varying electric and magnetic fields (1 Hz to 100 kHz). Health physics 2010;99(6):818–836.

Oster, G.. Phosphenes. Scientific American 1970;222(2):82–87.

Saunders, R.D., Jefferys, J.G.. A neurobiological basis for ELF guidelines. Health Physics 2007;92(6):596–603.

Schutter, D.J., Hortensius, R.. Retinal origin of phosphenes to transcranial alternating current stimulation. Clinical Neurophysiology 2010;121(7):1080–1084.

Setsompop, K., Kimmlingen, R., Eberlein, E., Witzel, T., Cohen-Adad, J., McNab, J.A., Keil, B., Tisdall, M.D., Hoecht, P., Dietz, P., et al. Pushing the limits of in vivo diffusion MRI for the human connectome project. Neuroimage 2013;80:220–233.

Szczepankiewicz, F., Westin, C.F., Nilsson, M.. Maxwell-compensated design of asymmetric gradient waveforms for tensor-valued diffusion encoding. Magnetic resonance in medicine 2019;82(4):1424–1437.

Taylor, P.C., Walsh, V., Eimer, M.. The neural signature of phosphene perception. Human brain mapping 2010;31(9):1408–1417.

Weintraub, M.I., Khoury, A., Cole, S.P.. Biologic effects of 3 tesla (T) MR imaging comparing traditional 1.5 T and 0.6 T in 1023 consecutive outpatients. Journal of Neuroimaging 2007;17(3):241–245.

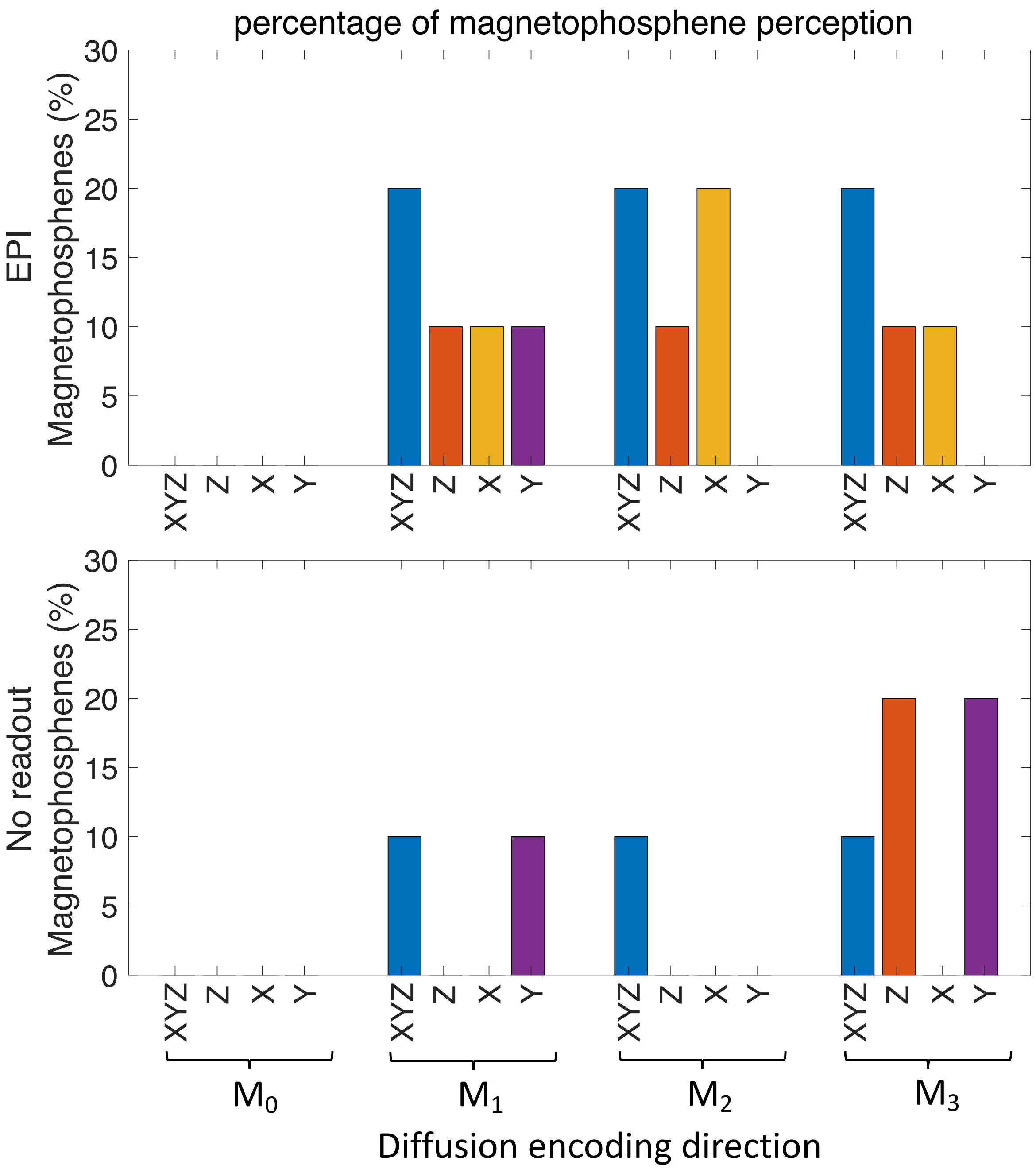

Figure S2 : The percentage of magnetophosphenes perception by the participants using different motion compensation (M_0, M_1, M_2, M_3) and readouts (EPI, and no readout).

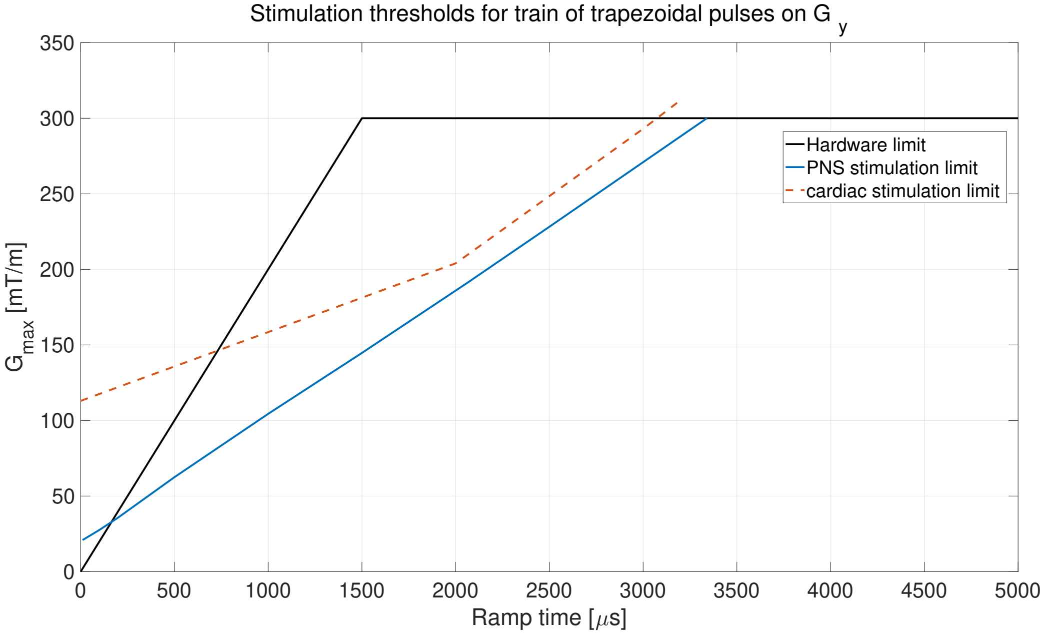

Figure S3 : The hardware limit, peripheral nerve stimulation, and cardiac thresholds for the G_y axis of Connectom gradient.

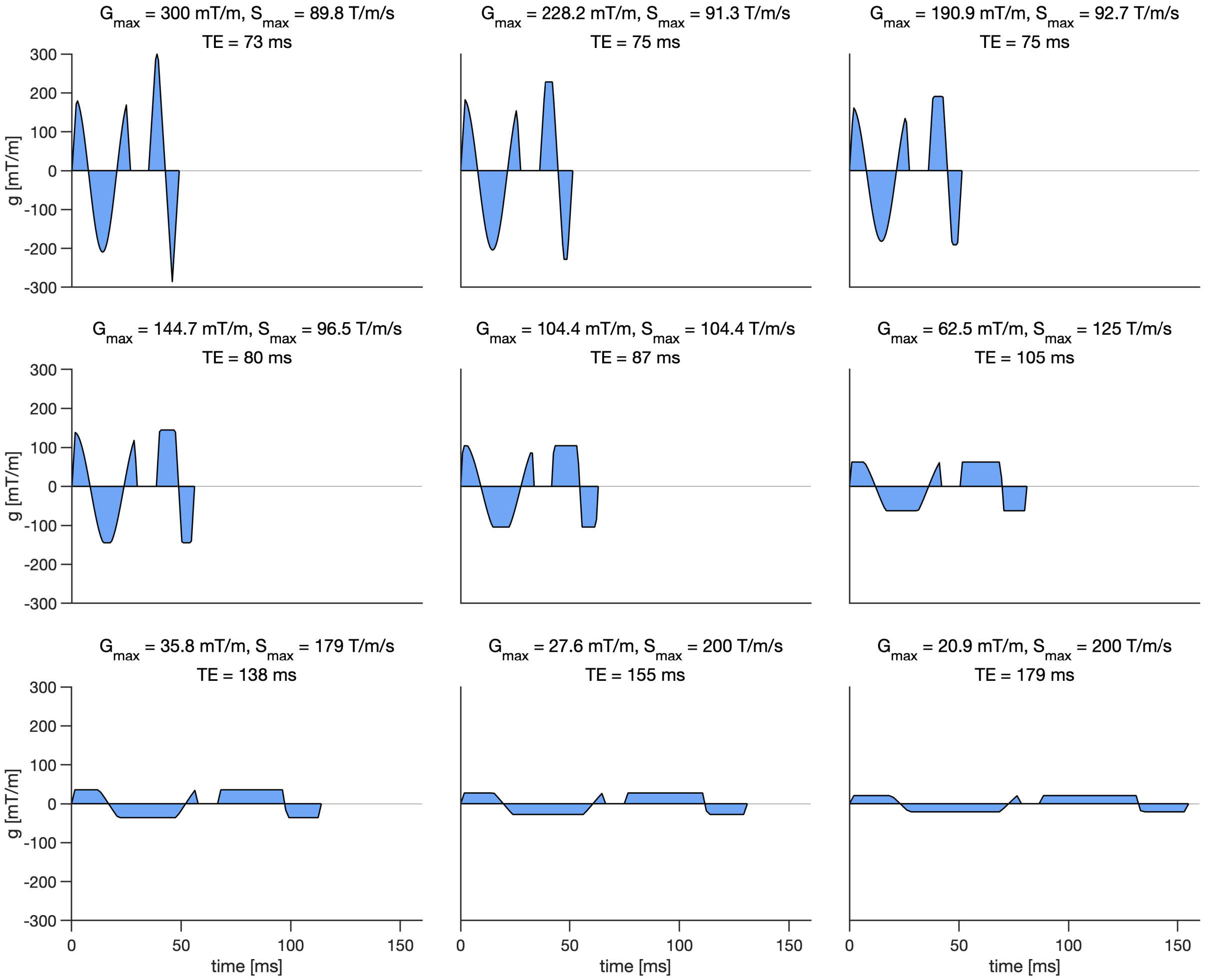

Figure S4 : Diffusion gradient waveforms designed for $b_{max} = 1000\,s/mm^2$ and motion compensation up to the second order (M_2) for different combinations of G_{max} and maximum slew rate (S_{max}) (provided by the vendor, Figure S3 and Table S1).

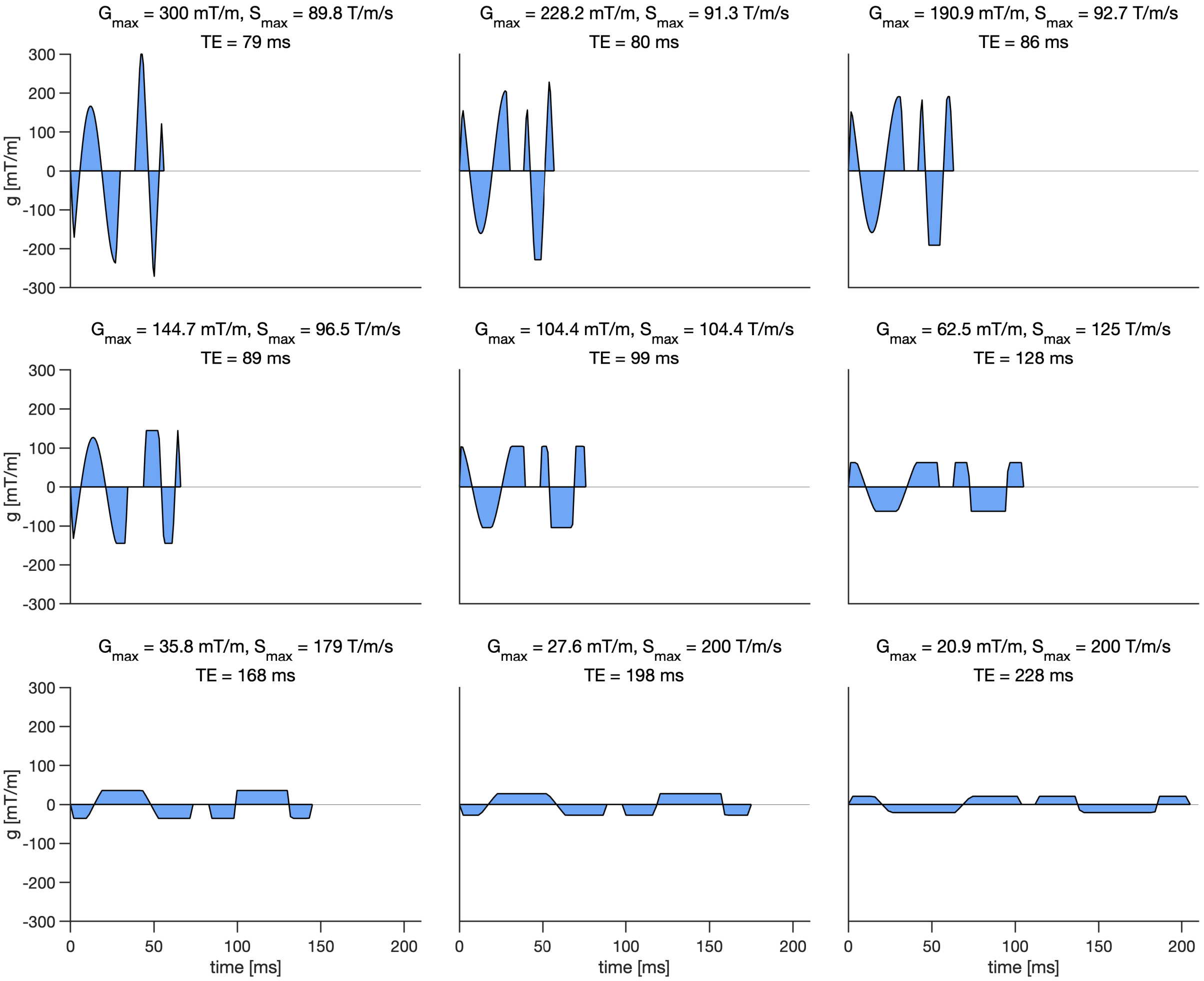

Figure S5 : Diffusion gradient waveforms designed for $b_{max} = 1000\,s/mm^2$ and motion compensation up to the third order (M_3) for different combinations of G_{max} and maximum slew rate (S_{max}) (provided by the vendor, Figure S3 and Table S1).

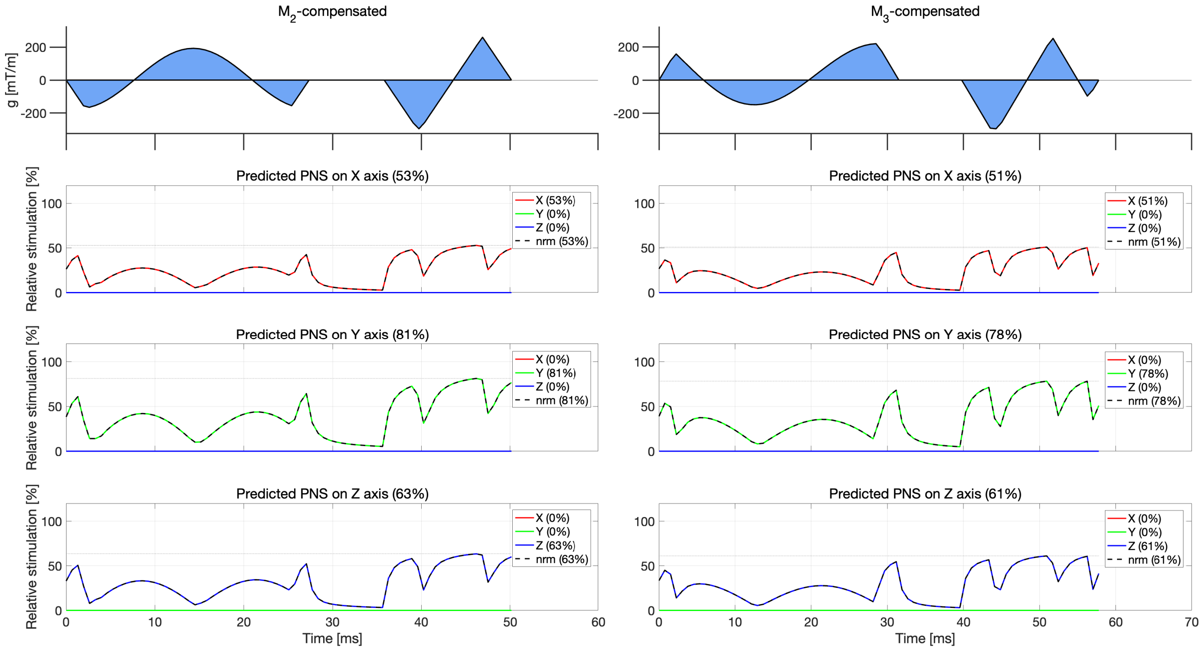

Figure S6 : Predicted PNS using SAFE model for the waveforms used in this study (shown on X, Y, and Z axes, it is clear that the Y-axis is the most restrictive axis for the PNS threshold).

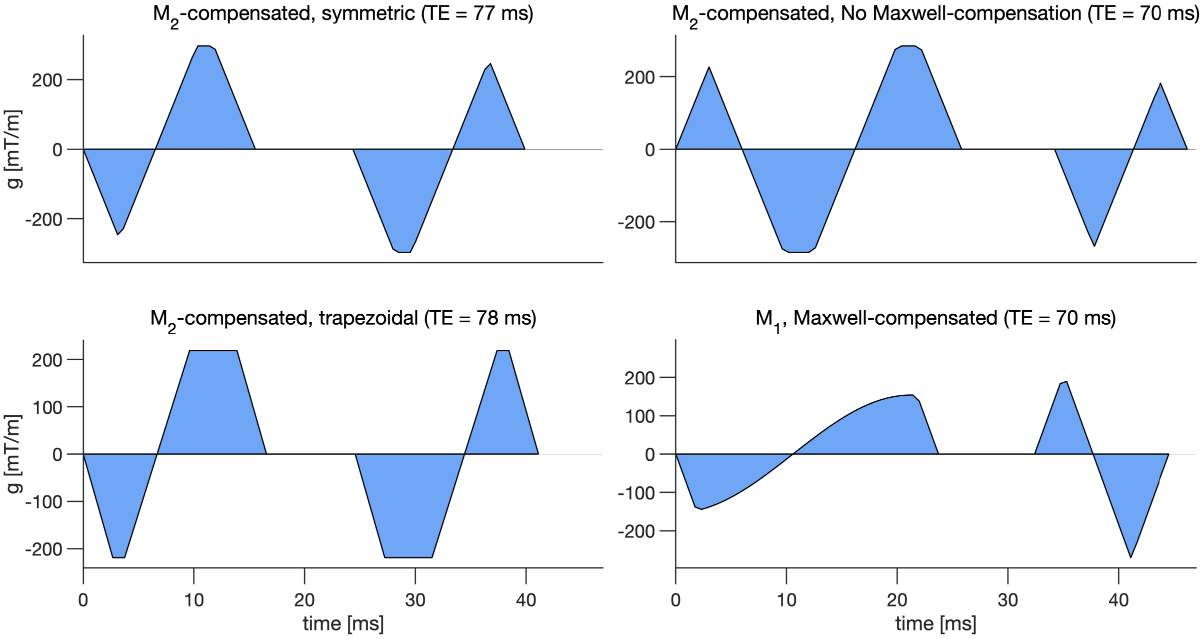

Figure S7 : M_2-compensated waveforms with different constraints and the corresponding TE.

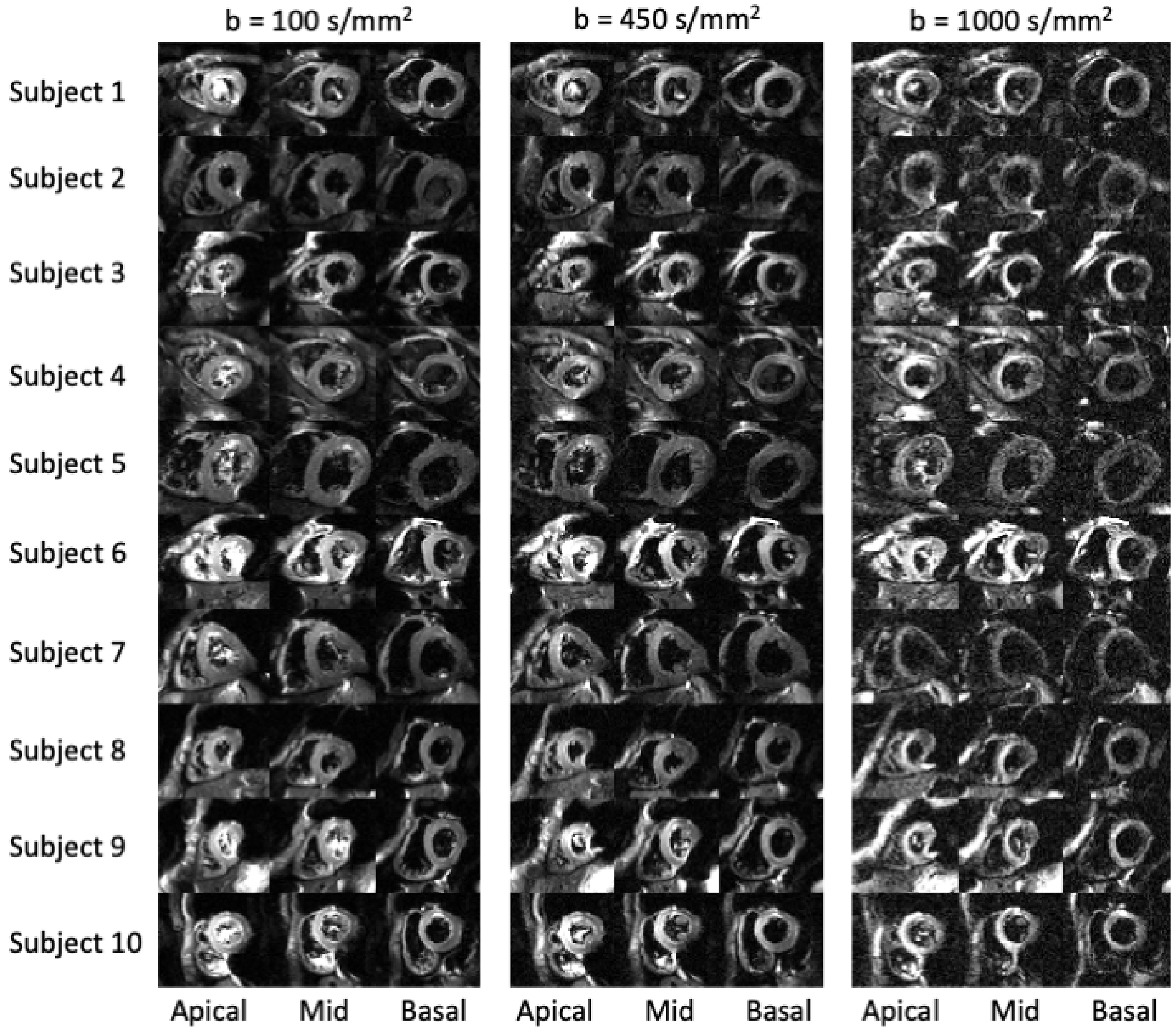

Figure S8 : Example diffusion weighted images acquired in a single direction (no averaging), with different b-values (100, 450, 1000 s/mm^2) for all 10 subjects.

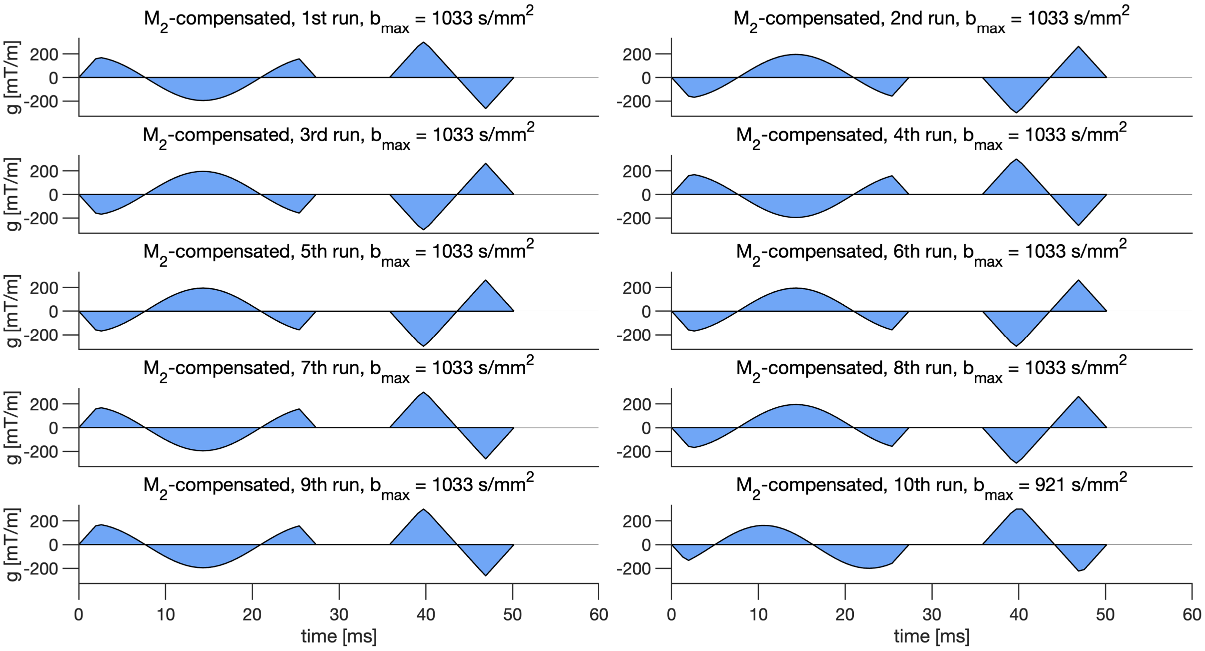

Figure S9 : Example diffusion gradient waveforms obtained from 10 consecutive run of the optimization algorithm (NOW toolbox)
