# Supplementary material for: In vivo diffusion MRI of the human heart using a 300 mT/m gradient system
Source: Magn Reson Med. Author manuscript; Available in PMC 2025 Mar 13. (PMC7617480; doi:10.1002/mrm.30118)
Supplement: Supporting Information Legand [file EMS203648-supplement-Supporting_Information_Legand.pdf]

SUPPORTING INFORMATION

Additional supporting information may be found in the online version of the article at the publisher's website.

Figure S1. Optimized motion compensated waveforms up to order three (M_0, M_1, M_2, M_3). The waveforms were

numerically optimized with the NOW toolbox to provide the shortest echo time for $b = 1000\ s/mm^2$, $G_{max} = 300\ mT/m$, and a maximum slew rate of 80 T/m/s.

Figure S2. The percentage of magnetophosphenes perception by the participants using different motion compensation (M_0, M_1, M_2, M_3) and readouts (EPI, and no readout).

Figure S3. The hardware limit, peripheral nerve stimulation, and cardiac thresholds for the G_y axis of Connectom gradient.

Figure S4. Diffusion gradient waveforms designed for $b_{max} = 1000\ s/mm^2$ and motion compensation up to the second order (M_2) for different combinations of G_{max} and maximum slew rate (S_{max}) (provided by the vendor, Figure S3 and Table S1).

Figure S5. Diffusion gradient waveforms designed for $b_{max} = 1000\ s/mm^2$ and motion compensation up to the third order (M_3) for different combinations of G_{max} and maximum slew rate (S_{max}) (provided by the vendor, Figure S3 and Table S1).

Figure S6. Predicted PNS using SAFE model for the waveforms used in this study (shown on x, y, and z axes, it is clear that the y-axis is the most restrictive axis for the PNS threshold).

Figure S7. M_2-compensated waveforms with different constraints and the corresponding TE.

Figure S8. Example diffusion weighted images acquired in a single direction (no averaging), with different *b*-values (100, 450, 1000 s/mm^2) for all 10 subjects.

Figure S9. Example diffusion gradient waveforms obtained from 10 consecutive run of the optimization algorithm (NOW toolbox).

Table S1. The maximum slew-rate (Smax) allowed by the system for each Gmax (provided by the vendor, Figure S3) with the corresponding minimum echo time for second- and third-order motion compensation (M2 and M3).

15222594, 2024, 3, Downloaded from https://onlinelibrary.wiley.com/doi/10.1002/mrm.30118 by Welsh Assembly Government, Wiley Online Library on [08/03/2025]. See the Terms and Conditions (https://onlinelibrary.wiley.com/terms-and-conditions) on Wiley Online Library for rules of use; OA articles are governed by the applicable Creative Commons License
